# 

**Published:** 1914-11

**Authors:** 


### CONTENTS.

ORIGINAL COMMUNICATIONS—

A Trip Through Europe and the Hospitals, by L. C. Fischer, M. D. .............................339

The Diagnostic Value of Examinations of the Spinal Fluid, by Allen H. Bunce, A. B., M. D. ...........354

Meeting of the Fulton County Medical Society, by R. R. Daly, M. D. .............................358

EDITORIAL .............................362

SELECTIONS AND ABSBTRACTS .................364

BOOK REVIEWS .............................390

